# Sub-threshold hypoalbuminemia, anemia, and occult infection trigger post-lobectomy bronchopleural fistula: a case report

**DOI:** 10.3389/fmed.2026.1774477

**Published:** 2026-05-07

**Authors:** Peng Dai, Haowei Wan, Rui Xu, Zichun Tang, Zongwei Xiao

**Affiliations:** 1 Department of Cardio-Thoracic Surgery, North Sichuan Medical College, School of Clinical Medicine, Nanchong, Sichuan, China; 2 Department of Cardio-Thoracic Surgery, West China School of Medicine, Sichuan University, Sichuan University Affiliated Chengdu Second People’s Hospital, Chengdu Second People’s Hospital, Chengdu, Sichuan, China

**Keywords:** bronchial-stump coverage, bronchopleural fistula, empyema, prognosis, VATS

## Abstract

A 56-year-old man presented with expectoration of blood clots (hemoptysis) and detached staples after undergoing left upper lobectomy plus wedge resection of the left lower lobe for lung cancer. Postoperative staple-line dehiscence is uncommon, occurring in approximately 1.5–3% of cases. When staples fail, the complication is frequently accompanied by bronchopleural fistula (BPF), a potentially catastrophic event that may determine empyema, aspiration pneumonia, and even erosion of major thoracic vessels with lethal hemorrhage. Morbidity and mortality from empyema are markedly higher in vulnerable populations such as the elderly, infants, and pregnant women. Current guidelines reserve prophylactic coverage of the bronchial stump for patients with clearly defined high-risk features—right pneumonectomy, neoadjuvant therapy, residual tumor at the stump, or diabetes mellitus—yet this policy may overlook individuals who harbor multiple, less overt risk factors. The possibility of BPF in patients with lower risk factors is not clearly evident in literature, and most studies focus on traditional high-risk populations. We report on a patient who possessed three such under-recognized predictors of BPF and ultimately developed complication despite standard surgical care.

## 1 Introduction

The powered stapler has become, in contemporary lung cancer surgery—whether lobectomy or wedge resection, the workhorse device for parenchymal and bronchial closure (1). Compared with hand-sewn sutures, these reloadable powered staplers reduce operative trauma, shorten operative time, simplify airway management, and lower overall morbidity (2, 3). For these reasons, they are now regarded as the gold-standard tool for bronchial transection in major lung resections. Nevertheless, staple-line failure, although uncommon, remains an ever-present threat. Published series report a post-lobectomy bronchopleural fistula (BPF) rate of 1.5–2.0% when staplers are used (4, 5), and once BPF occurs the associated mortality may reach 50% (6).

Risk stratification has identified several variables that magnify this BPF hazard: right-sided resections, neoadjuvant chemoradiotherapy, concomitant empyema or hemothorax requiring urgent pulmonary resection, an uncovered bronchial stump, microscopic tumor left at the stump, postoperative infection, and the aggregate burden of comorbidities such as diabetes, cardiovascular disease and hypoalbuminemia (6–12). Among these, diabetes mellitus is considered a high-risk factor due to impaired wound healing and microvascular complications (7, 10). Hypoalbuminemia and cardiovascular disease contribute to the aggregate burden of comorbidities that compromise tissue repair and oxygen delivery, though individually they may carry lower odds ratios than anatomical risk factors (9, 11, 12). To mitigate these dangers, current guidelines advocate bronchial-stump coverage (BSC) in selected high-risk patients, defined as those undergoing right pneumonectomy, receiving neoadjuvant therapy, having residual tumor at the stump, or with diabetes mellitus (7). The therapeutic corollary is that control of the septic focus—usually an underlying empyema—allows the fistulous tract to close spontaneously over time in cases of BPF (7, 13).

Hemoptysis is triaged by volume and velocity. Minor bleeding (blood-streaked sputum or <100 mL/24 h) responds to pharmacological haemostatics and supportive care (14, 15). Moderate hemoptysis (100–500 mL/24 h of pure blood) warrants aggressive medical therapy with immediate standby for bronchoscopic or endovascular intervention. Massive hemoptysis (>100 mL per expectoration, >500 mL/24 h, or any episode complicated by airway obstruction, respiratory failure or hemodynamic instability) usually requires emergent embolization or even surgical resection (14, 15).

BPF demands an integrated assessment of pleural sepsis, fistula size and location, patient performance status, and time elapsed since surgery. In clinically mild cases—low-grade cough, minimal sputum production, low-grade or absent fever—conservative measures are preferred: targeted antibiotics, nutritional optimization, adequate pleural drainage (when an air leak is documented), and bronchoscopic sealant techniques. Small defects can be closed successfully with fibrin glue or other biological adhesives, with reported success rates exceeding 70% (16, 17). Bronchoscopy plays a crucial role in BPF diagnosis by directly visualizing the bronchial stump defect, assessing fistula size and location, and enabling therapeutic interventions such as sealant application or stent placement (16, 17).

The lethality of BPF arises from the cascade triggered by a persistently patent airway–pleural communication. First, continuous soiling of the pleural space produces empyema; bacterial translocation then precipitates sepsis and septic shock—the leading cause of early death (18). Second, pus may be aspirated into the contralateral lung, resulting in aspiration pneumonia and acute respiratory failure. Finally, unchecked infection can erode major thoracic vessels, culminating in exsanguinating hemorrhage (18, 19).

## 2 Case presentation

### 2.1 First hospitalization (index surgery)

**Figure 1:**
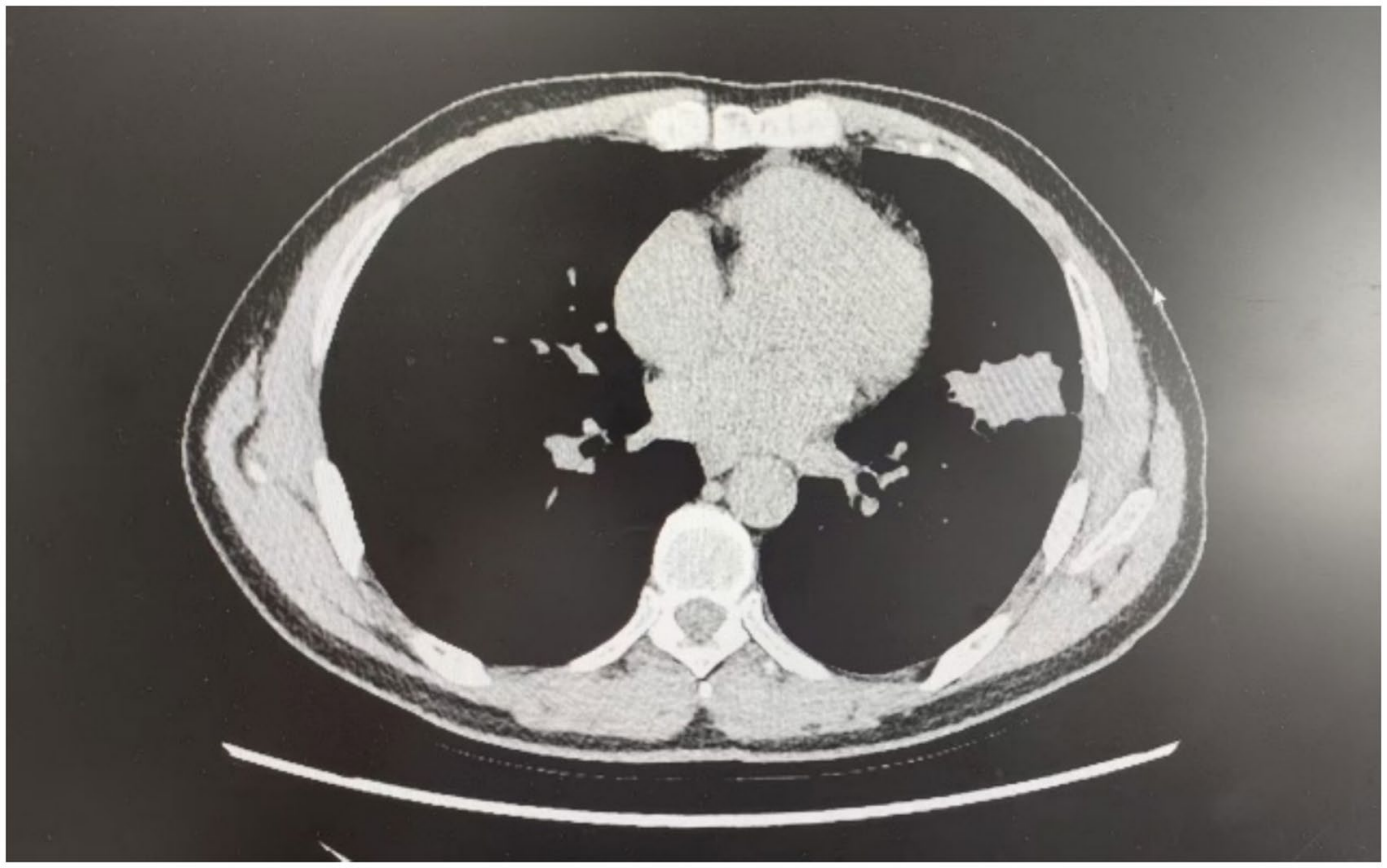
Pre-operative CT showed the dominant lesion in the left upper lobe, with an incidental nodule in the left lower lobe.

A 56-year-old Asian man (height 165 cm, weight 60 kg, Body Mass Index (BMI) 22.0 kg/m^2^) underwent video-assisted thoracoscopic left upper lobectomy plus wedge resection of the left lower lobe at our institution for a solid upper-lobe nodule (Figure 1). Four months prior, the patient was found to have a nodule in the left upper lobe with a maximum diameter of 20 mm during a physical examination. The patient was unwilling to undergo surgery and opted for conservative treatment. One month prior to surgery, a follow-up examination revealed enlargement of the left upper lobe nodule to a maximum diameter of 28 mm. After one week of anti-inflammatory treatment, no significant change in nodule size was observed. The patient decided to proceed with surgery.

We fully agree that histological diagnosis should take precedence over radical resection. However, the patient explicitly refused preoperative percutaneous biopsy and intraoperative frozen section examination after thorough informed consent discussion. The decision to proceed directly with lobectomy was based on the following clinical considerations: (1) rapid radiological progression of the pulmonary nodule; (2) failure of 2 weeks of empirical anti-inflammatory therapy; and (3) Computed Tomography (CT) features suggestive of malignancy. Given these high-risk characteristics and the patient’s desire to avoid staged procedures, a shared decision-making process ultimately led to the selection of radical resection.

Intra-operatively, the bronchus was divided with multiple VTR (Video-assisted Thoracoscopic surgery Reloadable) cartridges due to thickened bronchial wall and anatomical variation requiring sequential stapling: five VTR46G cartridges (45-mm staple line, 4.2 mm closed height), one VTR46W (2.5 mm), and one VTR46Y (3.8 mm); the lower-lobe wedge was secured with three additional VTR46W reloads. A 25 cm H₂O air-leak test sustained for 40 s was negative; therefore, no bronchial-stump coverage was applied.

Post-operative peak values were PCT 1.41 ng mL^−1^ and WBC 14.95 × 10^9^ L^−1^; histology revealed a benign lesion (inflammatory pseudotumor). He received cefuroxime, after which inflammatory markers normalized. A 3 × 2 cm furuncle at the level of the inferior angle of the scapula was incised and drained once leukocytosis had resolved. No oncological neoadjuvant or adjuvant therapy had been given.

Postoperative anticoagulation management included low-molecular-weight heparin (enoxaparin 4,000 IU subcutaneously) starting 12 h after surgery and continuing for 7 days, followed by oral rivaroxaban 10 mg daily for 2 weeks. Rivaroxaban was introduced as extended thromboprophylaxis following thoracic surgery guidelines, which recommend continuing prophylaxis for 2–4 weeks post-discharge in cancer patients and those with additional risk factors (age >60, major surgery, immobility). Although the final pathology was benign, the initial suspicion of malignancy and the patient’s age (56) justified extended prophylaxis to prevent venous thromboembolism during the recovery period. The patient did not receive therapeutic anticoagulation as he was ambulatory with low thromboembolic risk.

**Figure 2:**
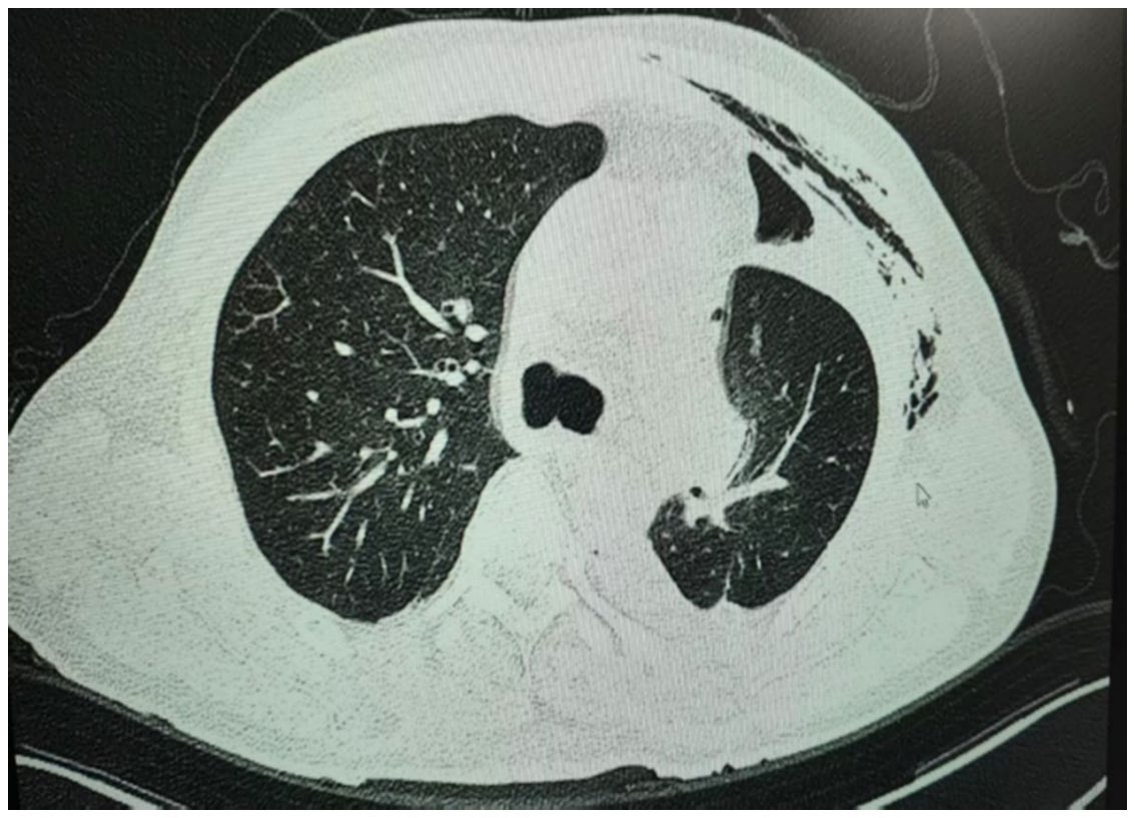
Postoperative computed tomography (CT) scan on day 2 after the surgery during the first hospitalization showed full expansion of the residual lung without evidence of pneumothorax or parenchymal compression.

On postoperative day 2, an enhanced chest CT scan was performed to confirm complete re-expansion of the residual lung and exclude early complications such as pneumothorax, pleural effusion, or hemorrhage, thereby guiding subsequent tube removal and discharge planning. The CT showed full expansion of the residual lung without evidence of pneumothorax or parenchymal compression (Figure 2). In the same day, the closed thoracic drainage was less than 50 mL, and the drainage tube was removed without any special discomfort. The patient remained hospitalized until postoperative day 5 for clinical observation, pain management optimization, and mobility assessment to ensure safe discharge despite having the drain removed on day 2. This 3-day observation period after drain removal is our standard protocol to monitor delayed complications such as recurrent pneumothorax, bleeding, or infection before discharge. The patient was discharged on postoperative day 5.

### 2.2 Second hospitalization (BPF episode)

**Figure 3:**
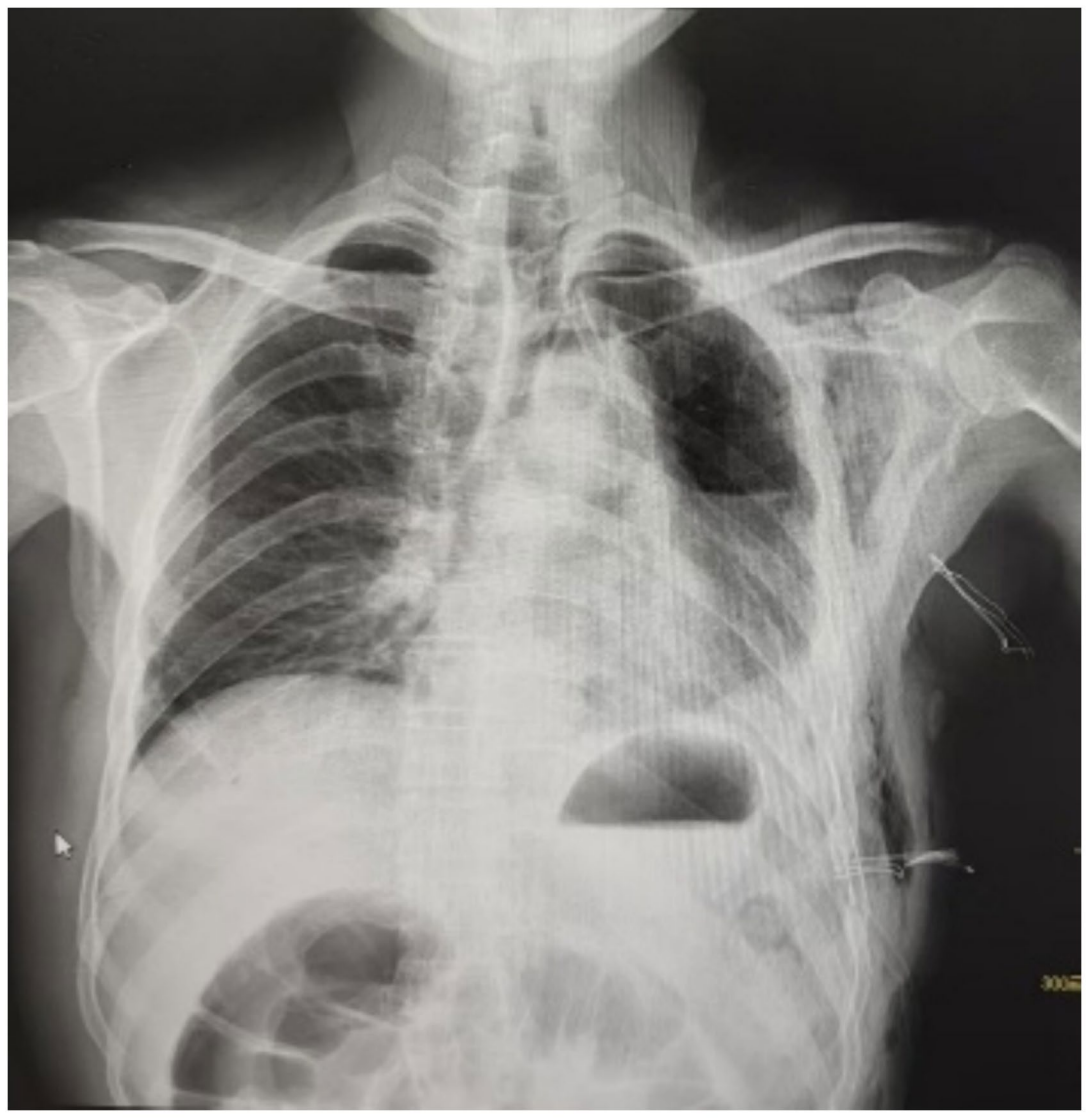
The patient’s second hospitalization chest radiograph revealed 40% compression of the left lung with moderate pneumothorax and pleural effusion.

Thirty-five days after the index surgery, the patient was readmitted with a 10-day history of hemoptysis. Initially he expectorated two 5-mL aliquots of blood daily; on day 8 he had a single moderate episode of 50 mL fresh blood mixed with clots, after which the frequency rose to 10 episodes per day while the volume per episode remained unchanged. On presentation he reported fatigue, poor appetite and pallor; laboratory studies showed hemoglobin 79 g L^−1^ (vs. 142 g L^−1^ preoperatively). He denied fever, but admission white-cell count was 11.14 × 10^9^ L^−1^ (vs. normal 4–10 × 10^9^ L^−1^) and procalcitonin 1.91 ng mL^−1^ (vs. normal <0.5 ng mL^−1^). The chest radiograph obtained on readmission revealed a 40% collapse of the left lung with a moderate hydropneumothorax (Figure 3), yet the patient remained asymptomatic from a respiratory standpoint with no dyspnea or tachypnea. For these reasons, chest tube insertion was deemed unnecessary.

### 2.3 Management and rationale

Given the moderate hemoptysis (estimated 100–200 mL/24 h) without hemodynamic instability, and the patient’s refusal of invasive procedures, we opted for conservative management with intravenous cefuroxime and pituitrin for 5 days, followed by 2 days of carbazochrome sulfonate alone. Bronchoscopy was discussed with the patient to confirm BPF and potentially apply sealant; however, the patient declined bronchoscopic examination due to anxiety and preference for noninvasive treatment. The hemoptysis abated promptly, and the patient was discharged home. No chest tube was inserted because: (1) the patient was clinically stable without respiratory distress; (2) the hydropneumothorax was moderate (30% collapse) and appeared chronic rather than acute; (3) the air leak was likely small given the patient’s ability to maintain normal respiration; and (4) the patient strongly preferred conservative management. The fistula likely healed spontaneously with antibiotic therapy and resolution of the septic focus. At discharge, chest X-ray showed persistent moderate hydropneumothorax with no significant change from admission, but the patient was asymptomatic.

### 2.4 Follow-up after discharge

We have established standardized protocols for prevention, early detection, and management. Intraoperatively, meticulous pleural closure and air leak testing are performed to minimize gas and fluid accumulation. For patients with chest tubes, the tube is removed when drainage decreases to less than 200 mL within 24 h with no air leak, and re-expansion of the lung is confirmed by imaging. Post-discharge, a structured follow-up protocol is implemented, with clinical assessments and chest imaging at 1 week, 1 month, and 3 months postoperatively. During follow-up, the focus is on evaluating respiratory symptoms, wound healing, and resolution of pleural abnormalities on imaging. This systematic monitoring mechanism ensures timely identification and management of delayed pneumothorax-effusion; however, no such complications occurred in this case during the 3-month follow-up period. This protocol aligns with current guideline principles: small asymptomatic pneumothorax may be managed conservatively, while larger or symptomatic pneumothorax require active intervention, including thoracentesis or chest drainage (20, 21).

Due to poor compliance with medical instructions, this patient did not undergo imaging re-examination as scheduled; we only assessed clinically that no respiratory symptoms occurred within 3 months after the second discharge. At 3-month follow-up, he remains symptom-free without respiratory complaints.

## 3 Elimination of the empyema cavity: surgical principles

This section is fundamental to the paper because it provides evidence-based rationale for our management approach and illustrates the spectrum of BPF treatment options. While our patient was managed conservatively, understanding the surgical principles of empyema cavity elimination is essential for clinicians to recognize when conservative management is appropriate versus when surgical intervention becomes necessary. This section also contextualizes why BSC is critical for prevention, as surgical cavity eradication is technically demanding and associated with significant morbidity.

The cornerstone of empyema therapy is physical eradication of the infected pleural space; only when the cavity is obliterated does suppuration cease and the BPF close. When BPF with consequent empyema occurs, the main objectives of treatment are the cleaning and sterilization of the empyematic pleural cavity and the re-closure of the bronchial stump (20, 21). Antibiotics alone achieve neither goal. Four classical mechanisms are used to collapse or fill the cavity: (1) open or closed drainage, (2) re-expansion of the lung, (3) thoracic wall collapse, and (4) obliteration with vascularized, pedicled tissue (20, 21).

When tube drainage fails to obliterate the space, surgical intervention is required. Pedicled muscle flaps—serratus anterior, latissimus dorsi, pectoralis major or intercostal—are mobilized, transposed into the hemithorax and secured over the closed bronchial stump (20, 21). A gas-tight seal buttressed by well-vascularized muscle separates airway pressure and secretions from the pleural surface, reducing the incidence of recurrent BPF to <5% (13). Flap bulk and pedicle length are deliberately maximized to guarantee robust local blood supply. If muscle is unavailable or insufficient, an omental pedicle based on the right gastro-epiploic arcade offers an excellent alternative, conveying both abundant vascularity and potent immunological activity (20, 21).

Not every patient, however, can tolerate formal cavity eradication. After pneumonectomy, especially in the frail or malnourished, the safest strategy is often controlled open drainage (Eloesser or Clagett window) followed by delayed closure once the mediastinum has stabilized and sepsis is quiescent—thereby restoring a “normal” post-pneumonectomy state without subjecting the patient to additional major reconstruction (20, 21).

In cases where surgical intervention is not immediately indicated, bronchoscopy plays a valuable role in conservative management. Beyond diagnosis, bronchoscopy enables therapeutic interventions such as fibrin glue application, sealant placement, or stent insertion to close small fistulous openings, with success rates exceeding 70% for defects <3 mm (16, 17).

## 4 Discussion

BPF is a multifactorial catastrophe that can be parsed into patient-related and external determinants. Host factors include malnutrition, hypoalbuminemia, diabetes-mediated impairment of wound healing, and neoadjuvant chemoradiation that renders bronchial tissue ischemic and infection-prone. Once local sepsis supervenes, bacterial collagenases corrode the staple line and surrounding cartilage, promoting abscess formation that eventually extrudes the staples into the airway and presents as expectorated metallic fragments.

External determinants are predominantly technical. Right-sided procedures, especially right lower-lobectomy or pneumonectomy, carry the highest BPF risk because the residual stump is short, relatively hypovascular and continually bathed by pleural fluid (16). Staplers are also less reliable when the bronchial wall is thickened, inflamed or anatomically foreshortened; these mechanical limitations have been quantified in recent bench-to-bedside studies (22).

Height and weight were used to calculate BMI (22.0 kg/m^2^), as malnutrition is a high-risk factor for bronchopleural fistula (23). According to the GLIM criteria, this patient did not meet the definition of malnutrition (23). This was one of the steps in calculating the number of low-odds ratio (OR) risk factors for this patient.

The resection was left-sided and the final pathology benign, excluding residual tumor at the stump. Nevertheless, three subtle but cumulative liabilities were present:

1. Moderate hypoalbuminemia (29 g L^−1^) on readmission, indicating impaired collagen synthesis and immune competence.
2. Chronic blood loss with resultant anemia (Hb 79 g L^−1^) that further compromised oxygen delivery to the bronchial margin.
3. An ongoing septic focus: a 2-cm furuncle over the scapular axis that yielded pus, plus borderline leukocytosis (11.14 × 10^9^ L^−1^) and procalcitonin 1.91 ng mL^−1^ on admission.

Furuncles are typically caused by *Staphylococcus aureus* and represent a common form of skin and soft tissue infection. This may indicate a history of bacterial infection or carrier status in the patient, which could increase the risk of BPF after thoracic surgery, as the pathological mechanism of BPF involves infection, necrosis, and fistula formation at the bronchial stump. Postoperative infection is one of the main precipitating factors for BPF. A furuncle is essentially a manifestation of bacterial infection and may suggest underlying infection history, immunocompromise, or bacterial colonization risk in the patient.

Although the exact temporal relationship between this low-grade infection and the first episode of hemoptysis cannot be established (antibiotics were taken irregularly), the constellation fulfils the “trend-to-sepsis” pattern now recognized as a BPF accelerator (9, 24, 25).

Multivariable modeling of 1,179 consecutive anatomical resections identifies seven independent predictors of BPF; their respective odds ratios are: right-sided surgery 6.00, ≥3 major comorbidities 3.04, female sex 0.42, positive bronchial margin 3.80, emergency surgery 6.91, post-operative adjuvant therapy 6.14, and absence of post-operative pulmonary infection 0.039 (9, 24, 25). Our patient possessed none of the high-impact variables yet still developed staple-line dehiscence—underscoring the continuum of risk and the concept that “lower-risk” does not equal “no risk.”

This observation highlights the Achilles heel of current prophylaxis: BSC is almost exclusively offered to patients who already carry obvious high-risk flags, whereas those in the intermediate grey zone are left uncovered. BSC employs well-vascularized flaps—intercostal muscle, pericardial fat pad, serratus anterior, latissimus dorsi or omentum—to isolate the bronchial stump from the contaminated pleural space, improving local perfusion and tensile strength (7). Randomized data demonstrate that universal BSC after any lobectomy reduces BPF from 2.8 to 0.7% with minimal added morbidity (7). Until such “all-comers” coverage becomes standard, surgeons should recognize that hypoalbuminemia, anemia and occult infection are sufficiently or in concert—to push an otherwise “low-risk” patient across the BPF threshold.

**Figure 4:**
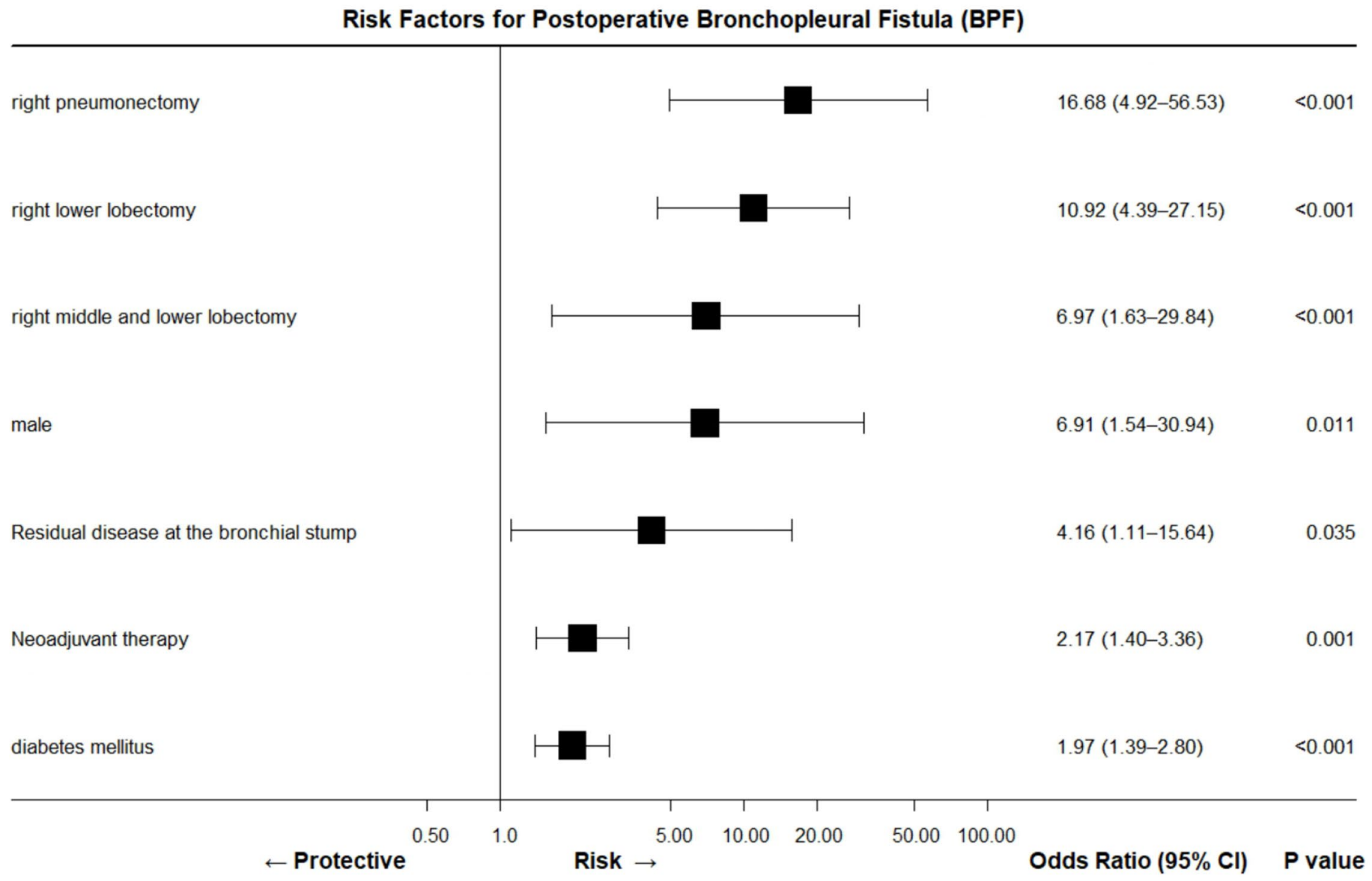
Forest plot summary of BPF risk factors (OR, 95% CI, *p*-value).

**Figure 5:**
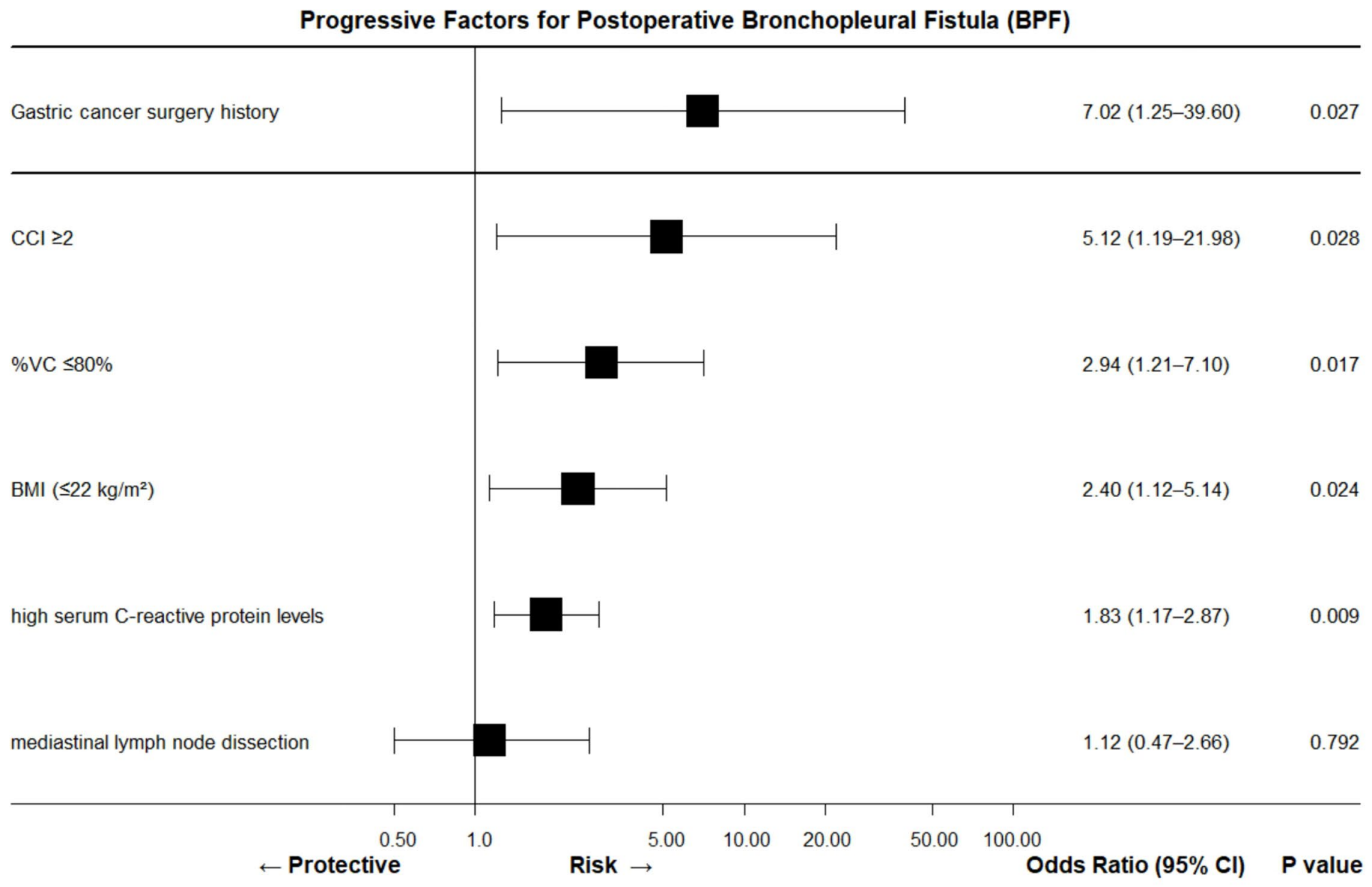
Forest plot summary of BPF progression risk factors (OR, 95% CI, *p*-value).

This case underscores BPF can occur even in the absence of “textbook” risk factors when several modest insults coincide. A 56-year-old man with left-sided, non-pneumonectomy resection for benign disease developed staple-line dehiscence after cumulative, low-grade threats—moderate hypoalbuminemia (29 g L^−1^), anemia and an inadequately treated skin focus of infection—overwhelmed wound healing. These individually “minor” variables, none qualifying as traditional BPF predictors, combined to breach the bronchial stump, illustrating that the *number* of sub-threshold factors may be as important as their severity (Figures 4, 5).

Consequently, we propose extending the indication for routine bronchial-stump coverage (BSC) beyond the usual high-risk scenarios (pneumonectomy, right lower lobectomy, stump tumor, neoadjuvant therapy, diabetes) to include any patient harboring two or more BPF-promoting elements such as albumin <30 g L^−1^, CRP > 10 mg L^−1^, BMI ≤ 22 kg m^−2^, %VC ≤ 80%, Charlson index ≥2, extensive nodal dissection, or age ≥60 years. This two-hit threshold, supported by recent multivariable data, is particularly critical for middle-aged and elderly patients whose limited physiological reserve magnifies the lethality of BPF once it occurs.

Middle-aged and elderly patients tolerate post-operative empyema poorly: they have longer stayed and higher 30-day mortality (26). Because BPF is the common gateway to this cascade, prevention is mandatory. Technical diligence—correct stapler height, preservation of bronchial blood supply and meticulous stump fashioning —must be coupled with rigorous correction of modifiable risk factors (hypoalbuminemia, anemia, infection) and early recognition of warning signs such as hemoptysis or unexplained anemia.

The single most effective prophylaxis is routine BSC with a vascularized flap, yet current practice reserves it for only the highest-risk anatomical or oncological scenarios (right pneumonectomy, neoadjuvant therapy, residual tumor, diabetes). We propose that any patient aged 50 years or older undergoing lung resection be offered BSC whenever two or more BPF-promoting factors—however “minor”—are present. This simple extension of indications, illustrated by the present case, can avert the vicious circle of BPF, empyema and septic shock in the very population least able to survive it.

## 5 Conclusion

This case illustrates that bronchopleural fistula can occur even after left-sided, non-pneumonectomy resection for benign disease when modest, under-recognized risk factors accumulate. Hypoalbuminemia, anemia, and an occult septic focus—none qualifying as traditional high-risk criteria—combined to breach the staple line. Current guidelines reserve bronchial-stump coverage for overt high-risk features; we advocate extending prophylactic coverage to any patient harboring two or more sub-threshold predictors, particularly middle-aged and elderly individuals whose limited reserve magnifies the lethality of BPF.

## Data Availability

The original contributions presented in the study are included in the article/supplementary material, further inquiries can be directed to the corresponding author.
